# A Hybrid Speller Design Using Eye Tracking and SSVEP Brain–Computer Interface

**DOI:** 10.3390/s20030891

**Published:** 2020-02-07

**Authors:** Malik M. Naeem Mannan, M. Ahmad Kamran, Shinil Kang, Hak Soo Choi, Myung Yung Jeong

**Affiliations:** 1 Department of Cogno-Mechatronics Engineering, Pusan National University, 2 Busandaehak-ro, 63 Beon-gil, Geumjeong-gu, Busan 609-735, Korea; naeem@pusan.ac.kr (M.M.N.M.); malik@pusan.ac.kr (M.A.K.); HCHOI12@mgh.harvard.edu (H.S.C.); 2 National Center for Optically-Assisted Ultrahigh-Precision Mechanical Systems, Yonsei University, Seoul 03722, Korea; snlkang@yonsei.ac.kr; 3 School of Mechanical Engineering, Yonsei University, Seoul 03722, Korea; 4 Division of Hematology/Oncology, Department of Medicine, Beth Israel Deaconess Medical Center and Harvard Medical School, Boston, MA 02115, USA

**Keywords:** Brain–computer interface, electroencephalography, eye tracker, hybrid BCI, canonical correlation analysis, steady-state visual evoked potentials, information transfer rate

## Abstract

Steady-state visual evoked potentials (SSVEPs) have been extensively utilized to develop brain–computer interfaces (BCIs) due to the advantages of robustness, large number of commands, high classification accuracies, and information transfer rates (ITRs). However, the use of several simultaneous flickering stimuli often causes high levels of user discomfort, tiredness, annoyingness, and fatigue. Here we propose to design a stimuli-responsive hybrid speller by using electroencephalography (EEG) and video-based eye-tracking to increase user comfortability levels when presented with large numbers of simultaneously flickering stimuli. Interestingly, a canonical correlation analysis (CCA)-based framework was useful to identify target frequency with a 1 s duration of flickering signal. Our proposed BCI-speller uses only six frequencies to classify forty-eight targets, thus achieve greatly increased ITR, whereas basic SSVEP BCI-spellers use an equal number of frequencies to the number of targets. Using this speller, we obtained an average classification accuracy of 90.35 ± 3.597% with an average ITR of 184.06 ± 12.761 bits per minute in a cued-spelling task and an ITR of 190.73 ± 17.849 bits per minute in a free-spelling task. Consequently, our proposed speller is superior to the other spellers in terms of targets classified, classification accuracy, and ITR, while producing less fatigue, annoyingness, tiredness and discomfort. Together, our proposed hybrid eye tracking and SSVEP BCI-based system will ultimately enable a truly high-speed communication channel.

## 1. Introduction

A brain–computer interface (BCI) provides a direct line of communication between a human brain and a computer by converting physiological signals into commands for the control of external devices [1,2,3,4,5]. BCIs are designed mainly to provide an alternative means of communication for people with severe motor disabilities [6,7,8,9]. Among a number of techniques, electroencephalography (EEG) is the most popular brain-imaging method for BCI implementation due to its noninvasive nature, low cost, portability and high temporal resolution [9,10,11,12,13]. In the literature, several BCI systems have been developed by using EEG signals, including [14] event-related desynchronization/synchronization (ERD/ERS) [3], steady-state visually evoked potentials (SSVEPs) [8,15], event-related potentials (ERPs) [16,17], and slow cortical potentials (SCPs) [18]. Among these, SSVEP-based BCIs are the most practical, because they support a large number of output commands and require little training time [19,20,21,22,23,24,25,26,27]. Recently, the SSVEP-based BCI has attracted increasing attention due to its high rate of communication and lack of any significant training requirement compared with other BCI systems [28,29,30,31]. Users of an SSVEP-based BCI are presented with a set of visual targets that are associated with possible characters, each of which flickers at a different, fixed frequency [32]. In an SSVEP BCI, target character/command where user is looking at is decoded by using corresponding SSVEP responses.

The use of various SSVEP-based BCI spellers for high classification accuracy as well as high communication rates has been proposed. Bin et al. reported, based on a six-target system, an information transfer rate (ITR) of 58 bits/min with an average accuracy of 95.3% [33], while Nakanishi et al. achieved an average accuracy and ITR of 92.78% and 91.68 bits/min with a 12-target system [34] and 91.35% and 166.91 bits/min with a 32-target system [35]. Yin et al., utilizing a 36-target SSVEP-based BCI speller, reported an ITR of 41.08 bits/min with an accuracy greater than 85% [36]; later on, by using hybrid P300 and SSVEP scores, they were able to achieve an average accuracy of 95.18% with an ITR of 50.41 bits/min [22]. Chen et al. reported an ITR of 105 bits/min for a 45-target BCI speller [27]; more recently, they introduced a high-speed spelling system with an average ITR of 267 bits/min and an average accuracy of 91.04% [28]. Nakanishi et al. developed a task-related component analysis technique to develop a high speed speller with an average accuracy of 89.93% and an average ITR of 325.33 bits/min [29]. In a recent study, Maye et al. proposed a multi-target SSVEP-based BCI system that only uses single flickering stimulus [37].

The stimulation frequencies ranging between 5 and 90 Hz could be used to elicit SSVEPs, but only few frequencies could be used due to the technological constraints of the current systems [38]. For instance, the conventional frequency coding method can only generate specific frequencies due to the limitation posed by the monitor refresh rate [39]. For example, a 60 Hz refresh rate monitor can only generate frequencies that are integer divisible of 60, e.g., 60/2 = 30, 60/3 = 20, and 60/4 = 15. Furthermore, Muller and co-authors showed that the detection of SSVEPs can be enhanced by using the harmonic components of the fundamental frequencies that are used to elicit SSVEPs, and, therefore, the use of harmonic frequencies should be avoided while designing SSVEP BCIs [40]. This further limit the available frequencies for a practical BCI system. For example, using 10 Hz as a fundamental stimulus frequency restricts the use of 20 and 30 Hz frequencies, since these are the harmonic components of 10 Hz. Moreover, many studies have shown that low frequency stimuli’s (4–12 Hz) can elicit strongest SSVEP responses; therefore, for high detection rates, stimulus frequencies should be selected from a low frequency range [41,42,43]. In the past, many researchers have proposed different stimuli designs to tackle these limitations and restrictions [28,44,45,46]. These methods include phase coding techniques [44,45], dual frequency stimuli [47,48], variable frame rate stimuli [49], multiple frequency sequential coding [50], amplitude modulation techniques [51,52], intermodulation frequencies [38,53,54], varying duty cycles [41,55], interpolation techniques [39], and joint frequency and phase modulation [28], but these techniques also have limitations. For instance, the number of phase lags are also dependent on the number of frames of the stimulus frequency in the phase tagging method, consequently resulting in a limited number of targets [38]. In the case of multiple frequency coding techniques, the number of targets increases as the cycle period increases, and the classification time window is proportional to the amount of stimulation frequencies that further decreases the overall BCI performance [38,50]. Furthermore, it has also been shown that low-frequency stimuli can cause photosensitivity-based epileptic seizures and high-levels of visual fatigue and discomfort, especially when presented with large number of targets and longer periods [21,43]. Many studies have used high-frequency stimuli and variable duty cycles to reduce visual fatigue and discomfort but at the cost of a decrease in the performance of SSVEP-based BCIs [21,43,56,57,58]. A few authors have also combined other EEG signals, e.g., P300, with SSVEP to overcome these limitations [22,59,60,61,62,63]. For instance, a 64 target BCI system was developed by using eight SSVEP frequencies and the P300 paradigm [22]. Though the number of targets can be increased by using only few frequencies, the incorporation of P300 increases the complexity of the task, which can also cause fatigue and a reduction in the classification accuracies.

On the other hand, researchers are combing several other modalities with EEG to enhance the performances of the current techniques. Recently, a bimodal approach that combined SSVEP with Electromyography (EMG) was developed to generate a 60 target speller with only 15 frequencies [64]. The speller was divided into four equal sections. The researchers instructed subjects to make fists (0–3) to identify the target section (1–4) and SSVEPs to detect target frequency. The data in this paper showed an average accuracy of 85.8% and an average ITR of 90.9 bits/min. Furthermore, eye tracking-based assistive technologies are emerging as an alternative tool to BCIs [65,66]. The development of modern camera systems and the increase of computing power has enabled the gathering of eye tracking data in real time, enabling the use of gaze as a control method for people with disabilities [67,68,69,70,71,72,73]. A comparison study of BCI, eye tracking, and electrooculography interfaces reported that participants found the BCI to be the easiest to use and eye tracking to be the least tiring [65]. They also showed that an SSVEP-based BCI performed better than an eye tracking-based speller when targets are densely located and small in size. Recently, eye tracking has been combined with EEG to remove ocular artifacts from EEG signals [74,75] and to develop hybrid BCI systems [65,76,77,78,79,80,81]. All these studies have shown improved performance as compared to EEG only, as well as the feasibility of combining EEG with eye tracking.

This paper proposes a combined EEG/eye tracking system for high-speed speller implementation in order to overcome the limitations described above; specifically (a) to improve practicality, especially on a computer screen (where only a limited number of flickering targets could be reliably implemented), and (b) a better user experience. Furthermore, the proposed speller identifies forty-eight targets by using only six frequencies by dividing speller into eight sub-matrices with six targets each and does so with an improved classification accuracy and an increased ITR. Eye-tracker data are employed to identify the target sub-matrix, while EEG data are used to identify the target frequency of the SSVEP. Moreover, the proposed speller reduces users’ visual discomfort, tiredness, annoyingness and fatigue, allowing for longer-duration use of the speller without any performance decrement; in contrast, a conventional BCI-speller (from now on referred to as a basic SSVEP speller) causes high fatigue and tiredness, which is a major problem that is responsible for performance decrement [43,52,82,83]. The proposed system is superior to basic SSVEP BCI-speller performances in terms of items classified, classification accuracy, and ITR. The comparison in the performances of the proposed, basic, and hybrid speller reveals the improved performance of the proposed hybrid approach. The significance of the performance improvement is statistically validated. In this study, a canonical correlation analysis (CCA)-based method is used to identify the target SSVEP frequency. In contrast to previous studies [34,83,84,85], the probability of misclassification of the targets with CCA is largely decreased by using six frequencies in the proposed hybrid approach. The methodological framework of the proposed system makes a truly high-speed communication channel possible.

## 2. Materials and Methods

### 2.1. Materials

#### 2.1.1. Proposed Hybrid SSVEP- and Eye-Tracking-Based Speller

**Figure 1:**
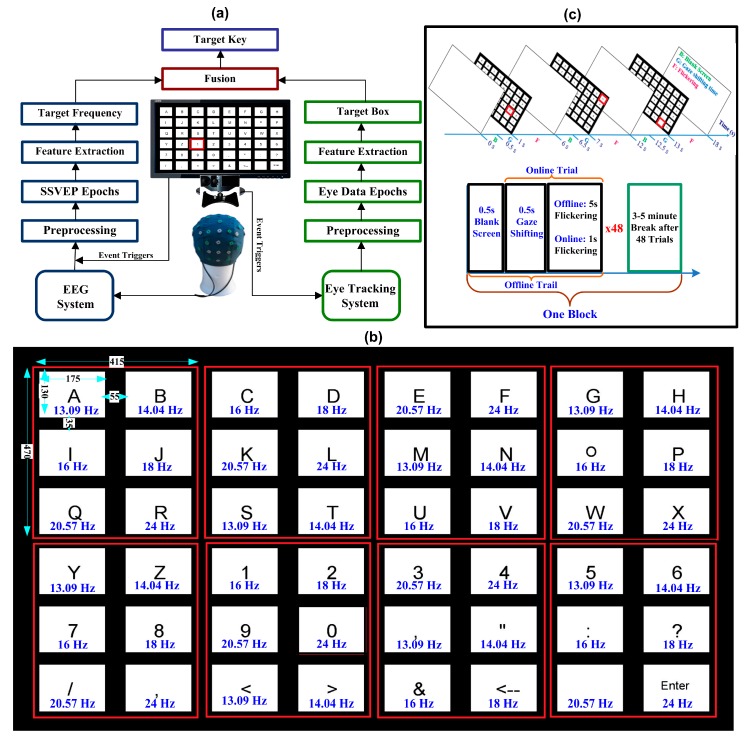
(**a**) Schematic diagram of the proposed framework. (**b**) Schematic diagram of the proposed speller with frequency of each target. Sizes were described in pixels. (**c**) Experimental paradigm.

The proposed hybrid eye-tracking and SSVEP BCI-based communication system operates as follows. While the user gazes at a target character flickering at a certain frequency, the SSVEP responses of the recorded EEG data are estimated and used as feature vectors. The target character that the user gazes at is identified by using the proposed framework illustrated in Figure 1a, which simply finds the frequency with the largest SSVEP response and locates the target box with the help of eye-tracking data. Once the target frequency and box are identified, the target letter is typed.

#### 2.1.2. Participants

Twenty healthy participants (male: 16, female: 4, aged 24–46 years) participated in this study. All had normal or corrected-to-normal vision. Only four had previous experience with an SSVEP-based BCI; the others were naïve to it. The experimental protocol was approved by the Institutional Review Board of Pusan National University. The experiment was conducted in accordance with the ethical guidelines established by the Institutional Review Board of Pusan National University and the Declaration of Helsinki. Each participant was asked to sign a written informed consent after being completely informed about the nature and purpose of the study. Each participant completed an offline experiment before performing the online experiments. To analyze and compare the effect of fatigue, tiredness, annoyingness, and discomfort, each participant performed experiments for a basic SSVEP speller on different days with a minimum gap of three days. During the experiments, all of the participants were seated in a comfortable armchair at a viewing distance of about 70 cm from the monitor. The experiment was conducted in a confined room with dim lighting to avoid environmental disturbance.

#### 2.1.3. Experimental Procedure

The new 48-target BCI speller, the core of the proposed high-speed communication system, uses only six frequencies. As shown in Figure 1b, the user interface is a 6 × 8 stimulation matrix containing 48 characters including the 26-letter Roman alphabet, 10 digits, and 12 other symbols. The matrix speller is divided into eight equal 3 × 2 sub-matrices, each containing six characters. Each character in each sub-matrix is assigned a different, fixed frequency. The eye tracker is used to identify the corresponding sub-matrix, specifically by tracking the user gaze, while EEG data are analyzed to identify the target frequency. A 24-inch LCD monitor (Asus, 144 Hz refresh rate) with a resolution of 1920 × 1080 pixels was used to present the speller. In this study, the frequencies used to flicker the targets were integer divisors of the monitor refresh rate, i.e., 144/11 = 13.0909, 144/10 = 14.40, 144/9 = 16.00, 144/8 = 18.00, 144/7 = 20.5714 and 144/6 = 24.00 Hz. A stimulus program was developed by using Psychophysics Toolbox Version 3 with MATLAB to generate visual stimuli. Event triggers were sent from the parallel port of the computer to the both EEG and eye tracking systems. 

#### 2.1.4. Offline Experiment

For each participant, the offline experiment with the 48-key speller consisted of three blocks. In each block, all 48 targets were presented randomly. Thus, each participant had three trials per character for a total of 144 (3 × 48) trials. Each trial, of 6s duration, started with a visual cue (a red square indicating a target stimulus) appearing on the screen for 0.5 s. The participants were advised to move their gaze to the target character as quickly as possible within the cue period. All the target characters started to flicker for 5 s right after the cue offset. Before the next stimulus, the screen was blank for 0.5 s after each trial. The subjects were instructed to avoid blinks during flickering periods. There was a rest for few minutes after each block. Figure 1c describes the experimental paradigm for the offline and online experiments.

#### 2.1.5. Online Experiment

In the online experiment, each trial was of only 1.50 s duration, including 0.5 s for gaze shifting and 1 s for stimulus flickering. The experiment was conducted in two stages, i.e., training and testing stage. There were three blocks in the training stage, each consisting of 48 trials to familiarize the subjects with online layout of the system. The testing stage included a cued-spelling task and a free-spelling task with three blocks each. In the cued-spelling task, targets were presented with a red box indicating a cue for the target character, whereas no cue was used in the free-spelling task. Each block in the cued-spelling task consisted of 48 trials. Auditory (a sound beep at the start of trial) and visual (red box on target stimuli) feedback was provided to the participants in real time. As soon as the target was classified by the online data analysis program, the identified target was typed into the text input field. In the free-spelling task, all participants were asked to input a 15-character sentence (“I LIVE IN KOREA”) without any cues. There was a 3 to 5-min break between two consecutive blocks.

#### 2.1.6. Control Conditions

After completing the experiments with the proposed speller, each participant performed experiments with basic and hybrid spellers from the literature. Each participant was given break of at least three days between each experiment.

##### Basic Speller

In this study, a forty-eight-target conventional speller with only SSVEPs was also implemented to compare the performance of the proposed system. The speller was developed by using Psychophysics Toolbox Version 3 with MATLAB. A conventional sinusoidal frequency coding scheme was used to generate flickering stimuli [86]. The frequency range for basic speller was 7–16.4 Hz (around alpha band) with a step size of 0.2 Hz.

##### Hybrid EEG-Eye Tacking

In this study, the proposed hybrid approach was also compared with a previously developed hybrid mental spelling system [81]. The basic idea of [81] was to divide the speller into three parts, i.e., left, middle, and right. In this sense, the misclassification of the SSVEPs could be reduced to improve the classification accuracies and ITR of the system. The number of frequencies that were used in this speller were equal to the number of targets. We implemented this speller with forty-eight targets with frequencies ranging from 7 to 16.4 Hz (around alpha band) with a step size of 0.2 Hz.

#### 2.1.7. Questionnaire

In addition to conventional comparison of classification accuracies and ITR, each subject also completed a questionnaire about how they felt after the experiment. The questionnaire included questions about the previous experience with BCIs, as well as their discomfort, fatigue and tiredness after the experiment. For each participant, the experiments with proposed and control conditions were performed on different days to counterbalance the settings.

#### 2.1.8. EEG Recordings

EEG data were recorded using a gUSBAmp with a 16-channel active electrode system that was developed by g.tec Medical Engineering, GmbH (Austria). Eight electrodes positioned over the parietal and occipital areas (PO7, PO3, POz, PO4, PO8, O1, Oz, and O2) were used to record the SSVEPs with a ground electrode FPz and reference electrode on right ear. All of the data were sampled at a rate of 1200 Hz. All the electrodes were positioned according to international 10–20 system. The impedance of all of the electrodes was reduced to below 1kohm. 

#### 2.1.9. Eye-Tracker Recordings

The eye movements were recorded with a video eye-tracking system (Eyelink 1000, developed by SR Research Ltd., Ottawa, ON, Canada). The sampling rate was 250 Hz. A velocity threshold of 30°/s was used to define the saccades; the acceleration threshold and minimum deflection threshold were 8000°/s^2^ and 0.1°, respectively. The eye tracker was calibrated for each participant.

### 2.2. Methods

In this study, eye tracking data and the CCA algorithm were used to identify the target sub-matrix and frequency of SSVEPs, respectively. Before analyzing data, eye tracking and EEG data were synchronized by using event triggers sent to both systems through the parallel port of the computer. In the offline and online experiments, data epochs were extracted according to event triggers that were produced by the MATLAB program. Given the latency delay in the visual system, the data epochs for the experiments were extracted in [0.14 s 5.14 s] and [0.14 s 1.14 s], respectively (time 0 indicated stimulus onset) [34]. To remove the common power line noise in EEG data, a notch filter of 50 Hz was applied to the data recording. In both the offline and online experiments, all of the epochs were first down-sampled to 300 Hz and then band-pass-filtered from 12 to 110 Hz. All the processing and analysis was performed in MATLAB.

#### 2.2.1. Sub-Matrix Detection

In this study, eye tracking data were used to identify the target sub-matrix in the proposed system. Eye tracker data were stored as pixels. Data epochs that were extracted using event triggers were used in this analysis. The mean of the gaze-direction data from each epoch was calculated as a feature to classify target sub-matrices.

#### 2.2.2. SSVEP Detection

CCA is a method for the extraction of similarities between two data sets [34,87]. CCA was first used in BCI studies by Lin et al. to detect SSVEP frequencies [88]. Considering two multidimensional variables *X*, *Y* and their linear combinations $x=X^{T}w_{x}$ and $y=Y^{T}w_{y}$, CCA finds the weight vectors, $w_{x}$ and $w_{y}$, that maximize the correlation between ***x*** and ***y*** by solving the problem:

(1) $$\rho (x,\;y)=\max_{w_{x},w_{y}}\frac{E[x^{T}y]}{\sqrt{E[x^{T}x]E[y^{T}y]}}=\max_{w_{x},w_{y}}\frac{E[w_{x}{}^{T}XY^{T}w_{y}]}{\sqrt{E[w_{x}{}^{T}XX^{T}w_{x}]E[w_{y}{}^{T}YY^{T}w_{y}]}}$$

The maximum of ρ with respect to $w_{x}$ and $w_{y}$ is the maximum canonical correlation. Projections onto $w_{x}$ and $w_{y}$ are called canonical variants. Here, *X* refers to a set of multi-channel EEG signals, and ***Y*** refers to the set of reference signals that have the same length as *X*. In SSVEP detection, the reference signals $Y_{k}\in {ℜ}^{2N_{h}\times N}$ are set as

(2) $$Y_{n}=\left[\begin{array}{l} \sin(2\pi f_{k}t)\\ \cos(2\pi f_{k}t)\\ \vdots \\ \sin(2\pi N_{h}f_{k}t)\\ \cos(2\pi N_{h}f_{k}t) \end{array}\right],\;t=\left[\frac{1}{f_{s}},\frac{2}{f_{s}},\cdots ,\frac{N}{f_{s}}\right]$$

where $f_{k}$ is the stimulation frequency, $f_{s}$ is the sampling frequency, $N_{h}=3$ is the number of harmonics, and ***N*** is the number of sample points. To recognize the frequency of the SSVEPs, CCA calculates the canonical correlation $\rho_{k}$ between the multi-channel EEG signals *X* and the reference signals at each stimulus frequency $Y_{k}$. The frequency of the reference signals with the maximal correlation is then selected as the frequency of the SSVEPs.

Once the target sub-matrix and frequency were identified, the corresponding character was selected as an output.

#### 2.2.3. Performance Evaluation

Classification accuracy and ITR were separately calculated for the offline and online experiments. The method for the calculation of ITR (in bits per minute, bpm) was [35]:

(3) $$ITR=\frac{1}{T}\left[log_{2}M+Plog_{2}P+(1-P)log_{2}\left(\frac{1-P}{M-1}\right)\right]\times 60$$

where *M* represents the total targets (i.e., 48 in this study), *P* represents the classification accuracy, and *T* represents the average selection time. In the offline experiments, the optimal BCI performance to calculate classification accuracy and ITR was estimated by using time windows between 1 and 4 s with an increment of 0.5 s. For the online experiment, the accuracy and ITR were calculated by using results obtained from the online data analysis program. In this study, the time windows that were used to calculate ITR for both the offline and online experiments also included the gaze shifting time (i.e., *T* = 0.5 s + 1 s = 1.5 s).

## 3. Results

In this paper, we propose a hybrid strategy to increase user comfort and to achieve high eye tracking and SSVEP-BCI-based speller classification accuracy and ITR.

### 3.1. Offline Data Analysis

**Figure 2:**
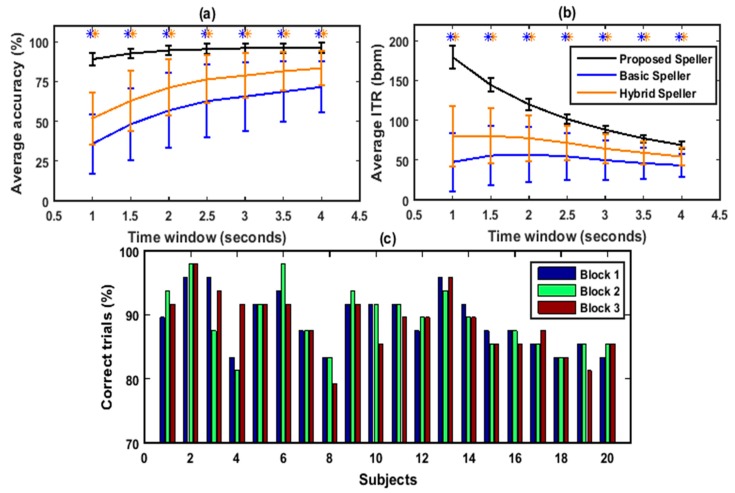
Performance comparison and evaluation of the proposed speller, the basic steady-state visually evoked potential brain–computer interface (SSVEP BCI)-speller, and the hybrid electroencephalography (EEG)-eye tracking-based speller for all subjects with different time windows during the offline experiment: proposed speller (black line), basic speller (blue line) and hybrid speller (orange line). (**a**) Average classification accuracies, (**b**) average information transfer rate (ITR), and (**c**) percentage of correct trials identified in each block for each subject. In (**a**,**b**), error bars indicate standard deviation. The asterisks indicate a significantly improved performance by the proposed speller (* *p* < 0.001).

To verify that the proposed framework performed better, the proposed approach was analyzed for the proposed 48-target speller in an offline experiment. Furthermore, the performance of the proposed speller was compared with the performance of a previously developed basic BCI-speller with SSVEPs only and hybrid EEG and eye tracking-based speller systems. Figure 2a shows a comparison of the average classification accuracies that were achieved by the proposed framework (black line), basic speller (blue line), and hybrid speller (orange line) for all subjects for epoch lengths ranging from 1 to 4 s with a fixed increment of 0.5 s; Figure 2b shows the corresponding average ITRs achieved by the proposed framework (black line), basic speller (blue line), and hybrid speller (orange line) with different epoch lengths. It could be seen that the accuracies and ITRs were higher for the proposed hybrid approach than those for the basic BCI-speller and the hybrid speller. Moreover, the significance of this performance improvement was statistically validated by using Mann–Whitney U test. The results of this statistical analysis verified that the performance improvement by the proposed hybrid approach was significantly higher than basic BCI-speller and hybrid speller for all time windows with *p* < 0.001. Additionally, in order to evaluate the discomfort, annoyingness, eye fatigue, and tiredness that was caused by the proposed, basic and hybrid speller systems, each subject was asked to fill in a questionnaire concerning his experience in the use of all speller systems after the experiments. The results of these questionnaire are listed in Table 1. It can be seen that 60% of the subjects felt a low level of annoyance due to flickering when using the proposed speller, whereas 55% of the subjects were highly annoyed when using the basic BCI-speller and the hybrid speller. Moreover, none of the subjects felt a high level of eye fatigue after using the proposed BCI-speller, while 65% of the subjects were highly fatigued after using both of the previously developed spellers. Furthermore, all the subjects were also asked about the overall tiredness they felt after using all these spellers. It is noteworthy that none of the subjects felt tiredness above a medium level after using the proposed speller, whereas 75% of the subjects were highly tired after using the other spellers. According to the results, all of the subjects were significantly more comfortable using the proposed speller system as compared with the spellers used in previous BCI studies [34,39]. Thus, the proposed speller could be implemented as a more comfortable and easy-to-use mode for practical and clinical applications, e.g., patients in locked-in state [89]. Since the average accuracy with the proposed speller was relatively higher (>89.03%) with all of the epoch lengths, the highest ITR was obtained with the shortest epoch length. According to Nakanishi et al. [35] and Equation (3) when using longer-length data, a minor increase in the classification accuracy leads to a significant decrease of ITR. For example, compared with 1 s data length, classification accuracy increased by 3.65% when using the 2 s data length (89.02% vs. 92.67%); however, the ITR dropped from 179.60 to 144.32 bpm. Correspondingly, in an online system that used the proposed framework, the 1 s epoch length was found to be optimal for the achievement of a high ITR, and there was no overlapping in the epochs. Figure 2c shows the percentage of the correct trials that were identified in each block by the proposed framework for each subject. Table 2 shows all of the subjects’ classification accuracies and ITR for an epoch length of 1 s.

### 3.2. Online Data Analysis

This study evaluated the proposed BCI speller while using two online cued- and free-spelling tasks. Table 3 lists the classification accuracy and ITR for all subjects cued-spelling tasks in the training and testing sessions. The average accuracy in the cued-spelling task in training session was 89.72%, which resulted in an average ITR of 181.90 bpm across all subjects. In the testing session, the average accuracy and ITR were 90.35% and 184.06 bpm, respectively. The online classification accuracy and ITR were slightly higher than those obtained in the offline experiment (accuracy: 89.03% vs. 90.35%; ITR: 179.60 bpm vs 184.06 bpm; Table 2 and Table 3). This could have been due to the familiarization with the proposed hybrid approach achieved by the subjects in the training sessions. Across individuals, the minimal and maximal ITR were 162.57 bpm (subject 19) and 206.98 bpm (subject 13), respectively. Table 4 shows the results of the free-spelling tasks. After some practice sessions for familiarization with the speller layout (without any cue), all of the subjects successfully completed the tasks. For subjects 7, 11, 15 and 18, the stimulus time was increased to 1.25 s to improve the classification accuracy; for subjects 4, 8 and 19, the gaze-shifting time was increased to 1 s due to the difficulty in rapidly shifting their gaze. The mean ITR achieved by the system was 190.73 bpm (minimum: 159.23 bpm (subjects 4, 8 and 19); maximum: 212.31 (subjects 6, 10 and 13)). An average spelling rate of 35.79 characters per minute (cpm) was achieved by the proposed system with maximum of 39.11 cpm. The overall results showed that there were no significant differences in the ITR of the cued- and free-spelling tasks.

## 4. Discussion

User comfortability plays an important role in the performance of BCI systems. As discussed in previous studies, in basic BCI spellers, the flickering of a large number of frequencies causes eye fatigue and discomfort for patients, thus rendering concentration on specific targets difficult, especially after using the speller for long period of time [43,52,82,83]. In the past, the necessity of decoding a large number of frequencies remained the key obstacle to the improvement of BCI-based spelling systems’ classification accuracy and ITR. Certainly, classification-accuracy and ITR shortcomings are precisely the problems that make the practical implementation of BCI spellers so difficult. However, recent advances in SSVEP-based BCIs have developed techniques and algorithms to overcome these obstacles. Several studies have proposed different stimulus design techniques to realize large number of targets with only few frequencies, but they have also suffered limitations [21,38,43,50,57,58,90]. Furthermore, a few researchers combined SSVEPs with P300 to generate more targets with less frequencies, but this was at the cost of task complexity, which eventually affected the performance of the system. In order to overcome these issues, the present study tested a hybrid approach that combines EEG and eye-tracking systems to not only reduce user discomfort but to also to achieve high classification accuracies and ITRs. The proposed framework for the implementation of the 48-target BCI speller uses only six frequencies. It is noteworthy that this is only 15% of the frequency number that is used in any BCI speller study to decode 36 or more commands to date [21,28,63]. The present BCI speller achieved an average high spelling rate of 184.06 bpm in the cued-spelling task, and an even higher rate, 190.73 bpm, in the free-spelling task. Table 5 lists the comparison of the recently developed SSVEP-based BCI systems including the proposed approach. In this comparison, the number of electrodes used to record SSVEP, the number of commands presented, the number of frequencies used to decode these commands, the accuracy and ITR are utilized as performance evaluation metrics. To the best of our knowledge, the ITRs that were achieved by the proposed hybrid approach are not highest ever achieved, but they are among the highest achieved with any speller system (Table 5). For further comparison, the mean ITR of a code-modulated visually evoked potential (cVEP)-based system was 116.4 bpm, the mean ITR of an SSVEP-based speller was 87.50 bpm, and the mean ITR of a P300-based speller was 17.4 bpm [28].

The present significant performance improvement can be attributed to the novel speller design that was implemented by combining the EEG and eye-tracking approaches.

The present study demonstrates the feasibility of a comfortable and high-speed speller that can achieve an ITR of up to 212.31 bpm. It should be noted here that only four subjects were familiar with the SSVEP-based BCI speller and layout. It has been reported in BCI literature that the major challenge in SSVEP-based spellers is to find a tradeoff between accuracy and ITR [20,28,52]. The selection of the time window has a high impact on the accuracy and ITR [95,96]. Many previous studies have already reported that CCA-based classification has high errors when using a short time window [34,83,84,96]. This might be due to the fact that decoding large number of frequencies (i.e., the number of frequencies that are equal to number of targets) may cause the production of errors in the classification of SSVEP targets. In contrast, the proposed hybrid approach uses only six frequencies to decode forty-eight targets. Furthermore, the performance of the proposed BCI-speller has been compared with the performance of a conventional SSVEP-based basic BCI-speller and a previously developed hybrid EEG and eye tracking speller. The results of this comparison revealed a significant improvement in the performance by using proposed approach (Figure 2a,b). A Mann–Whitney U test was used to statistically validate this performance improvement, and the results of this analysis showed that the proposed speller is significantly better in terms of both accuracy and ITR for all time windows with *p* < 0.001. Another advantage of the proposed framework is the considerably lower probability of target-key misclassification relative to the previous BCI speller systems that use a number of frequencies equal to the number of targets (e.g., 40 targets [28]), particularly those systems that also use short-duration EEG data (e.g., 1 s) (Figure 2). As discussed above, another potential advantage is that the proposed speller has considerably low annoyingness, fatigue and tiredness as compared to the basic BCI-speller (Table 1). This can also be attributed to the use of only six frequencies, and this could also have made it possible to use the proposed speller for longer period of time which is highly difficult to do with basic BCI-spellers. In contrast, the basic speller uses forty-eight frequencies for forty-eight targets, which causes a high level of discomfort and fatigue. It was also indicated by previous studies that the simultaneous flickering of a large number of stimuli can cause discomfort and fatigue to users, and this can also affect the performance of the system. Furthermore, another important advantage of the proposed framework is that it can overcome the restrictions and limitations that are caused by the monitor refresh rate to generate large number of frequencies to decode large number of targets [34,38,39], since the proposed speller only uses six frequencies that can be generated by any monitor. In the light of the above, the proposed BCI-speller system could be used as an efficient and better alternative to the previous speller systems.

**Figure 3:**
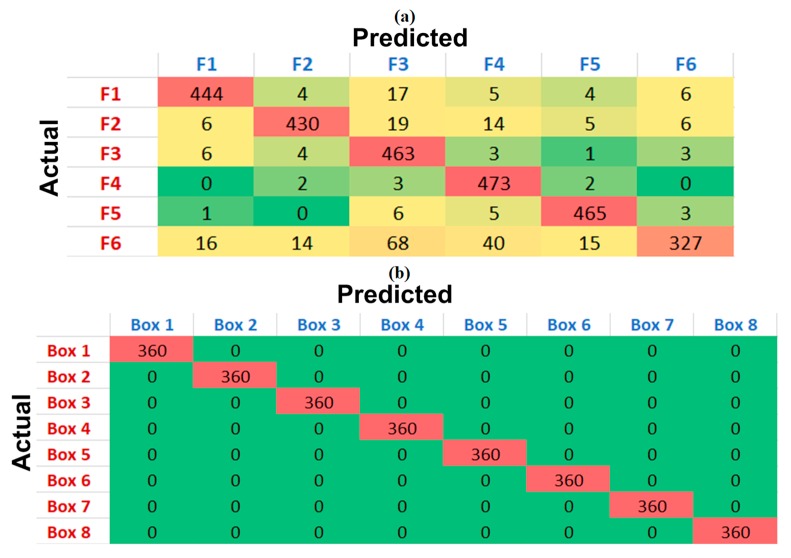
Confusion matrices. (**a**) SSVEP predictions. (**b**) Eye tracking predictions.

Since the main aim of this study was to propose a novel speller design for SSVEP-based BCI spellers, the proposed speller nonetheless has room for improvement. First, phase information could be added to the stimuli for more efficient target-frequency detection. Nakanishi and co-authors [20] used different phase values to better discriminate the target frequency from the recorded SSVEP signals as compared with a conventional frequency coding scheme. Additionally, the accuracy of the proposed system could be improved by using more enhanced SSVEP detection algorithms. Additionally in this regard, the algorithm that was utilized for target-frequency detection could be improved by incorporating filter bank analysis and individual training data. In filter bank analysis, all EEG data could be divided into different frequency sub-bands to improve the classification of the SSVEP targets. Chen and co-authors [84] illustrated the use of filter bank analysis to enhance the performance of conventional CCA-based target detection. Further improvements in the classification accuracy could be achieved by replacing conventional reference signals by individual training data for each target frequency. Individual training data for each target could be recorded before the actual experiment and could be used instead of sine/cosine reference signals. Yuan and co-authors [83] showed that inter-subject information could be used to update the reference signals to improve the detection of the target frequency in SSVEP-based BCIs. Moreover, the selection of flickering frequencies can be further optimized to enhance the performance of the proposed system. Figure 3 shows the confusion matrices for SSVEP and eye tracking predictions, which can be helpful in selecting optimal parameters for the proposed speller. It can be seen that eye tracking predictions had no errors, as the size of the target box was large enough to be detected correctly. This is consistent with the previous studies which have shown that eye tracking classifications are low if the targets are densely located with small sizes [65]. In contrast, the SSVEP predictions showed misclassifications, specifically for frequency six. The prediction errors with this frequency highly affect the overall classification accuracy and ITR of the system. Therefore, the optimization of the frequency selection could highly increase the performance of the proposed system. Furthermore, higher ITRs could be achieved if the stimulus duration was separately optimized for each subject. Indeed, system parameters such as electrode locations, the number of electrodes, stimulation frequencies, the effect of frequencies from different SSVEP ranges, and the number of trials for templates could be optimized for each individual to achieve the best system performance [97]. Therefore, it is our immediate future plan to study the effect of all these parameters on the performance of the proposed system and to select optimal parameters with best performance.

It is important to mention here that, whereas eye-tracker-based spellers’ spelling rate is 5 to 10 words per minute [28,98], they require a high sampling rate as well as a high level of user concentration on the target (i.e., “gaze control” without any movement), which is difficult for most people to manage [65,99]. Furthermore, the equipment needed for such eye tracking in real time is, at least at present, expensive, which makes it impractical for application [100]. One may argue that the Eyelink used in this study is also a research grade eye tracking system and can achieve good accuracy by itself. However, we argue on the basis of previous studies that have used cheap cameras for eye tracking and have shown that eye tracking classifications are low if the targets are densely located with small sizes and SSVEP-based BCI performs better in such a scenario [65,81]. On the other hand, there are higher chances of misclassification of targets in SSVEP-based BCIs when decoding a large number of targets. Therefore, combining eye tracking with an SSVEP-based BCI can provide a good solution that can be used to achieve best results. In other words, the present study used eye tracking to detect large boxes (a region covered by six keys, as shown in Figure 1b) that can be easily detectable by any ordinary camera, and that, therefore, do not require exceedingly high levels of concentration and motionlessness. Thus, the proposed hybrid framework can be considered for employment as an optimal speller not only in many BCI applications but also in many other applications like artifact rejection from EEG data.

## 5. Conclusions

This paper presents a hybrid framework to implement a novel speller design to reduce user discomfort and to increase the classification accuracy and ITR of a speller system by combining eye tracking and an SSVEP BCI with stimulus frequencies ranging from 13 to 24 Hz. The proposed speller is superior to most of the spellers that have been developed in the literature in terms of user comfortability, items classified, classification accuracy, and ITR. The high point of the proposed speller is that it only uses six frequencies to classify forty-eight targets, whereas a basic speller uses a number of frequencies that is equal to the number of targets. Furthermore, the discomfort, fatigue, annoyingness and tiredness caused by the proposed speller are lesser as compared to the basic and hybrid spellers. A comparison with the basic and hybrid spellers revealed a statistically validated significantly better performance of the proposed framework.

## Figures and Tables

**Table 1:** Results from the questionnaires.

Speller	Experience with SSVEP BCI		Flickering Annoying			Eye Fatigue			Level of Tiredness				
	Yes	No	Low	Medium	High	Low	Medium	High	1	2	3	4	5
Proposed	**4**	**16**	12	8	0	12	8	0	7	10	3	0	0
Basic			0	9	11	0	7	13	0	0	5	10	5
Hybrid EEG and eye tracking			0	8	12	0	8	12	0	0	4	11	5

The numbers represent number of subjects. The level of tiredness was evaluated on a scale of 1 to 5: 1 = not tired; 2 = little tired; 3 = moderately tired; 4 = tired; and 5 = highly tired.

**Table 2:** Classification accuracy and information transfer rate of proposed framework with 1 s epoch lengths in offline experiment.

Sub	Classification Accuracy (%)	Information Transfer Rate (bpm)
**1**	91.67	188.34
**2**	97.22	209.89
**3**	92.36	190.84
**4**	85.42	167.03
**5**	91.67	188.34
**6**	95.14	201.38
**7**	87.50	173.88
**8**	81.94	156.02
**9**	89.58	180.96
**10**	92.36	190.85
**11**	90.97	185.84
**12**	88.89	178.59
**13**	95.14	201.38
**14**	90.28	183.40
**15**	86.11	169.28
**16**	86.81	171.58
**17**	86.11	169.28
**18**	83.33	160.35
**19**	84.03	162.57
**20**	84.72	164.78
**Mean**	**89.03**	**179.60**
**SD**	**4.224**	**14.728**

**Table 3:** Classification accuracy and information transfer rate for the online cued-spelling task.

Sub	Training Session		Testing Session	
	Classification Accuracy (%)	Information Transfer Rate (bpm)	Classification Accuracy (%)	Information Transfer Rate (bpm)
**1**	89.58	180.97	88.19	176.21
**2**	95.13	201.38	95.13	201.38
**3**	90.97	185.84	92.36	190.85
**4**	87.50	173.88	86.81	171.57
**5**	92.36	190.85	93.06	193.42
**6**	95.83	204.15	95.83	204.15
**7**	88.19	176.22	89.58	180.97
**8**	86.80	171.57	86.11	169.28
**9**	92.36	190.85	93.06	193.42
**10**	93.75	196.02	93.06	193.42
**11**	88.89	178.58	91.67	188.33
**12**	91.67	188.33	92.36	190.85
**13**	95.83	204.15	96.53	206.98
**14**	88.89	178.58	91.97	185.84
**15**	85.42	167.02	87.50	173.88
**16**	90.97	185.84	90.28	183.39
**17**	85.42	167.02	86.81	171.57
**18**	86.81	171.57	86.11	169.29
**19**	82.64	158.19	84.03	162.57
**20**	85.42	167.02	87.50	173.88
**Mean**	**89.72**	**181.90**	**90.35**	**184.06**
**SD**	**3.788**	**13.298**	**3.597**	**12.761**

**Table 4:** Classification accuracy and information transfer rate for the online free-spelling task.

Sub	Trial Length (s) (Gaze Shift + Stimulus)	Total No. of Trials (Correct/Incorrect)	Spelling Rate (cpm)	Information Transfer Rate (bpm)
**1**	1.5 (0.5 + 1)	45 (41/4)	36.44	186.34
**2**	1.5 (0.5 + 1)	45 (43/2)	38.22	203.03
**3**	1.5 (0.5 + 1)	45 (43/2)	38.22	203.03
**4**	2.0 (1 + 1)	45 (44/1)	29.36	159.23
**5**	1.5 (0.5 + 1)	45 (43/2)	38.22	203.03
**6**	1.5 (0.5 + 1)	45 (44/1)	39.11	212.31
**7**	1.75 (0.5 + 1.25)	45 (43/2)	32.76	174.02
**8**	2.0 (1 + 1)	45 (44/1)	29.36	159.23
**9**	1.5 (0.5 + 1)	45 (43/2)	38.22	203.03
**10**	1.5 (0.5 + 1)	45 (44/1)	39.11	212.31
**11**	1.75 (0.5 + 1.25)	45 (44/1)	33.52	181.98
**12**	1.5 (0.5 + 1)	45 (42/3)	37.33	194.45
**13**	1.5 (0.5 + 1)	45 (44/1)	39.11	212.31
**14**	1.5 (0.5 + 1)	45 (43/2)	38.22	203.03
**15**	1.75 (0.5 + 1.25)	45 (44/1)	33.52	181.98
**16**	1.5 (0.5 + 1)	45 (43/2)	38.22	203.03
**17**	1.5 (0.5 + 1)	45 (42/3)	37.33	194.45
**18**	1.75 (0.5 + 1.25)	45 (43/2)	32.76	174.03
**19**	2.0 (1 + 1)	45 (44/1)	29.36	159.23
**20**	1.5 (0.5 + 1)	45 (42/3)	37.33	194.45
**Mean**	**-**	**-**	**35.79**	**190.73**
**SD**			**3.47**	**17.849**

**Table 5:** Comparison of the present study with recent SSVEP-based BCI studies.

Study	Stimuli	Multimodal	Frequency Range	NE	NC	NF	Average Accuracy (%)	Information Transfer Rate
Present	Rectangles	Yes	Mid	8	48	6	90.35 (84.03–96.53)	190.73 (159.23–212.31)
Nakanishi et al. [29]	Rectangles	No	Low	9	40	40	89.83 (79.50-97.50)	325.33 (263.00–376.58)
Chen et al. [84]	Rectangles	No	Low	9	40	40	91.95 (78.50–99.50)	151.18 (114.48–175)
Chen et al. [28]	Characters	No	Low	9	40	40	91.00 (77.00–99.50)	267.0 (199.8–315.0)
Bin et al. [33]	Rectangles	No	Low	9	6	6	95.30 (83.30–100.0)	58.00 (40.00–67.00)
Kwak et al. [91]	LED	No	Low	8	5	5	91.30 (81.40–98.60)	32.90 (19.60–51.00)
Müller -Putz et al. [92]	LED	No	Low	4	4	4	72.50 (44.00–88.00)	19.70 (4.10–34.20)
Chen et al. [27]	Characters	No	Low	9	45	45	88.70 (73.30–98.90)	61.0 (45.00–75.00)
Martinez et al. [93]	Checkerboard	No	Low	6	4	4	96.50 (82.30–100.0)	29.60 (17.00–38.70)
Min et al. [94]	Line-grid	No	Low	3	6	6	42.50 (20.00–63.30)	3.20 (0.10–9.40)

NE: number of electrodes, NC: number of commands, and NF: number of frequencies. Frequency range: low = 4–12, mid = 12–30, and high, >30.

## Data Availability

The dataset used and/or analyzed during the current study are available from the corresponding author upon reasonable request.
